# ATP Antagonizes Thrombin-Induced Signal Transduction through 12(S)-HETE and cAMP

**DOI:** 10.1371/journal.pone.0067117

**Published:** 2013-06-24

**Authors:** Jaione Burzaco, Manuel Conde, Luis A. Parada, José L. Zugaza, Jean-Paul Dehaye, Aida Marino

**Affiliations:** 1 Department of Biochemistry and Molecular Biology, Faculty of Science and Technology, University of the Basque Country, Bilbao, Spain; 2 Instituto de Patología Experimental, Universidad Nacional de Salta, Salta, Argentina; 3 Department Genetics, Physical Anthropology and Animal Physiology, Faculty of Science and Technology, University of the Basque Country, Bilbao, Spain; 4 Achucarro Basque Center for Neuroscience, Bizkaia Science and Technology Park, Zamudio, Spain; 5 IKERBASQUE, Basque Foundation for Science, Bilbao, Spain; 6 Biochemistry and Cellular Biology Laboratory, Institute of Pharmacy C.P. 205/3, Université Libre de Bruxelles, Brussels, Belgium; Center for Cancer Research, National Cancer Institute, United States of America

## Abstract

In this study we have investigated the role of extracellular ATP on thrombin induced-platelet aggregation (TIPA) in washed human platelets. ATP inhibited TIPA in a dose-dependent manner and this inhibition was abolished by apyrase but not by adenosine deaminase (ADA) and it was reversed by extracellular magnesium. Antagonists of P2Y_1_ and P2Y_12_ receptors had no effect on this inhibition suggesting that a P2X receptor controlled ATP-mediated TIPA inhibition. ATP also blocked inositol phosphates (IP1, IP2, IP3) generation and [Ca^2+^]_i_ mobilization induced by thrombin. Thrombin reduced cAMP levels which were restored in the presence of ATP. SQ-22536, an adenylate cyclase (AC) inhibitor, partially reduced the inhibition exerted by ATP on TIPA. 12-lipoxygenase (12-LO) inhibitors, nordihidroguaretic acid (NDGA) and 15(S)-hydroxy-5,8,11,13-eicosatetraenoic acid (15(S)-HETE), strongly prevented ATP-mediated TIPA inhibition. Additionally, ATP inhibited the increase of 12(S)-hydroxy-5,8,10,14-eicosatetraenoic acid (12(S)-HETE) induced by thrombin. Pretreatment with both SQ-22536 and NDGA almost completely abolished ATP-mediated TIPA inhibition. Our results describe for the first time that ATP implicates both AC and 12-LO pathways in the inhibition of human platelets aggregation in response to agonists.

## Introduction

Activation of human platelets is a key event in the processes of hemostasis and thrombosis. Several agonists including ADP, thrombin, and thromboxane A_2_ (TXA_2_) can activate platelets [1]. These agonists affect platelets leading to shape change, aggregation, or promoting that the granule release their content [2]. Thrombin is a serine protease which is activated by extrinsic and intrinsic coagulation cascades at the vascular injury site. It is not only a coagulation enzyme catalysing the conversion of soluble fibrinogen into an insoluble fibrin clot, but also an extremely important agonist for platelet activation [3]. Thrombin primarily mediates cellular effects through protease-activated receptors (PARs). Three of the four PARs known (PAR1, PAR3 and PAR4) are activated by thrombin with PAR1 and PAR4 being present in human platelets. Both receptors are coupled to a G_αq_subunit [4].

ADP is released during platelet activation, becoming a critical molecule in hemostasis. ADP also cooperates with other molecules, including thrombin, to potentiate many platelet responses [5]. Two different P2 receptors, P2Y_1_ and P2Y_12_, involved in the ADP-induced platelet responses have been cloned. The P2Y_1_ receptor mediates PLC activation via a G_αq_ subunit and subsequently regulates intracellular calcium ([Ca^2+^]_i_) mobilization and platelet shape changes [5]. P2Y_12_ receptor, on the other hand, is coupled to the G_αi_ subunit, which prevents the activation of AC, whereupon the intracellular cAMP concentration decreases. P2Y_12_ receptor behaves as a negative regulator of platelet activation [6]. The P2Y_12_-dependent G_αi_ activation also potentiates the release of granule contents [7] and can directly activate the α_IIb_β_3_ integrin via phosphoinositide-3 kinase [8]–[11].

ADP-induced platelet aggregation requires coactivation of P2Y_1_ and P2Y_12_ receptors [12]. Thrombin and thrombin receptor-activating peptides (TRAPs) have been shown to activate both G_αq_ and G_αi_ pathways [13] but unlike ADP, thrombin alone is unable to activate both pathways [14]. Glycoprotein Ibα and ADP act synergistically to amplify the PAR1- but not the PAR4-coupled responses [15]. Thrombin not only requires secreted ADP and P2Y_12_ activation to stimulate G_αi_ and activate PAR1 via G_αq_ but also, at high concentrations, it can regulate PAR4 pathway [16]. It has been described that ticagrelor and other cyclopentyltriazolopyrimidines (P2Y_12_ antagonists) selectively block the ADP component in the thrombin response resulting in a potent inhibition of platelet activation whereas they are ineffective for P2Y_1_ [17].

ATP and ADP are present in platelets at approximately equimolar concentrations [18] and extracellular ATP inhibits ADP-induced platelet activation, since it acts as a competitive antagonist through P2Y_1_ and P2Y_12_ receptors [19]. It has been reported that ATP stimulates P2X_1_ receptor in human platelets and increases the intracellular calcium concentration without generating platelet aggregation [20]. Moreover, studies on transgenic animals showed that P2X_1_ receptors play an important role in platelet activation, particularly under conditions of shear stress and thus during arterial thrombosis [21]. Besides, this receptor could be involved in the aggregation of human platelets induced by collagen [22].

ATP and other nucleotides such as, GTP, GDP or GDP-β-S inhibit both thrombin- and ADP-mediated platelet activation [23]. TIPA and the inhibition of the cellular secretion mediated by ATP is accompanied by a decrease in [Ca^2+^]_i_ mobilization, this suggests that an extracellular P2X-like site could be responsible for the effects of these nucleotides [23]. Dragan and Ellis found that thrombin-untreated cells**,** extracellular ATP, GTP and AMP increased the 12(S)-HETE production. ATP activated 12-LO by an unknown mechanism and increased by 3-fold the 12(S)-HETE formation. A purinergic binding site is proposed to activate this pathway [24].

The aim of this work was to examine the interaction between extracellular ATP and platelets exposed to thrombin. Our results suggest that AC and the 12-LO pathways are implicated in the inhibition of TIPA mediated by ATP. This physiological inhibition of human platelets in response to strong agonists is mediated by a combined action between the P2Y_12_ receptor and the inhibition of the intracellular levels of 12(S)-HETE.

## Materials and Methods

### Reagents

Adenosine 3′, 5′ biphosphate (A_3_P_5_P), fibrinogen, acid citrate dextrose, ADA, ADP, ATP, α,β-methylene ATP, βγ-methylene ATP, benzoyl ATP, 2 methylthio ATP, apyrase, ethylenediaminetetraacetic acid (EDTA), ethylene glycol-bis(β-aminoethylether)-*N*,*N*,*N*′,*N*′-tetraacetic acid (EGTA), N-[2-hydroxyethyl] piperazine-N′-[2-ethanesulfonic acid] (HEPES), 15(S)-HETE, 3- isobutyl-1-methylxanthine (IBMX), milrinone, dipyridamole, NDGA, protein kinase, 3′, 5′-cyclic AMP dependent (PKA), polyethylenimine, sodium nitroprusside (SNP) and thrombin were obtained from Sigma-Aldrich Chemical (St. Louis MO, USA). Fura-2 AM was from Molecular Probes (Eugene OR, USA). Myo-[2-^3^H]inositol was from GE Healthcare (Barcelona, Spain). Dowex AG1-X8 column was from Bio-Rad Laboratories (Hercules PA, USA). Trichloroacetic acid (TCA) was purchased from Panreac (Barcelona, Spain). 1H-[1], [2], [4]oxadiazolo[4,3-a]quinoxalin-1-one (ODQ) was from Alexis Biochemicals (San Diego CA, USA), 9-(tetrahydro-2-furyl)adenine (SQ-22536) was obtained from (Calbiochem-Novabiochem Corporation (San Diego CA, USA). 12(S)-HETE levels were measured using an EIA kit according to the manufacturer’s instructions (Assay Designs, Ann Arbor MI, USA).

### Platelet Aggregation Measurement

Platelets were acquired from the Blood Bank at the Galdakao Hospital in Spain. Platelet aggregation was carried out as described previously [25], [26]. Briefly, washed human platelets were resuspended in HEPES buffer (10 mM HEPES pH 7.4, 145 mM NaCl, 5 mM KCl, 10 mM Glucose); apyrase free at 2.5×10^8^ cells/ml, aliquoted in 500 µl and placed in siliconized glass cuvettes and prewarmed at 37°C without stirring. The samples were then placed into a thermostatted aggregometer at 37°C (Menarini model Aggrecorder II PA-3220) and stirred before treatment. Aggregation was measured as the percentage of the maximum change in light transmission against a buffer blank for 10 min. All assays were performed in the presence of 1 mM CaCl_2_.

### [Ca^2+^]_i_ Mobilization

[Ca^2+^]_i_ levels were monitored and calculated as described [25], [27]. Briefly, washed human platelets at 2.5×10^8^/ml in a nominally Ca^2+^-free standard medium (no added chelator or CaCl_2_; pH 7.4) were loaded with 1 µM Fura-2 AM and 0.018 U/ml apyrase grade V at 37°C for 45 min. Subsequently, acid citrate dextrose was added (2% vol/vol; pH 6.5), and cells were washed by centrifugation (350×g, 20 min, 20°C). Finally, cells were resuspended in fresh standard medium; apyrase-free at 2×10^8^ cells/ml. Cell aliquots (0.5 ml) were transferred to a cuvette and prewarmed at 37°C for 5 min.

Fluorescence was measured with a spectrofluorimeter (SLM Aminco Bowman Series2, SLM) equipped with a thermostated cell holder and a magnetic stirrer. Fura-2 fluorescence was monitored continuously using monochromator settings of 340 and 380 nm (excitation) and 505 nm (emission). These experiments were performed either in the presence of 1 mM CaCl_2_ or without external Ca^2+^ but with 2 mM EGTA-K_2_-H_2_.

### [^3^H]inositol Phosphates Generation

Measurement of [^3^H]inositol phosphates was carried out as described [25]. Briefly, washed human platelets were incubated with myo-[2-^3^H]inositol at 37°C for 3 hours. Subsequently, cells were washed and resuspended in HEPES buffer (pH 7.4) at 6–8×10^8^ cells/ml and then allowed to rest for ≥25 min before experimentation. Platelet suspensions (0.5 ml) were prewarmed at 37°C for 5 min followed by placing them into the aggregometer (37°C) and stirred. After stimulation, reaction was stopped by adding 0.5 ml ice-cold 10% (vol/vol) perchloric acid. Samples were centrifuged at 1,000×g for 5 min at 4°C, and the supernatants were neutralized with 1.5 M KOH/75 mM HEPES and recentrifuged at 1,500×g for 5 min at 4°C. The supernatant was diluted with 10 mM HEPES (pH 7.4)/2 mM EDTA. The [^3^H]inositol phosphates were separated by anion-exchange chromatography on a Dowex AG1-X8 column as described [25], [26]. The radioactivity was measured in a liquid scintillation counter Tri-Carb (model 2700 TR Series; Packard Instrument Company, Meriden, CT).

### cAMP Accumulation

Washed human platelets at 4×10^9^ cells/ml were aliquoted in 350 µl and placed into the aggregometer in a siliconized glass cuvettes warmed at 37°C and stirred before treatment. In order to prevent the rapid degradation of cAMP by the well known phosphodiesterases type II, III and V present in platelets [28], cells were preincubated with a mixture of specific inhibitors (1 mM IBMX, 10 µM milrinone and 50 µM dipyridamole) for 5 min. Then, cells were incubated in the presence of agonists at different times. After stimulation, the reaction was stopped by the addition of ice-cold 10% TCA. Samples were kept on ice for 30 min and centrifuged at 21,000×g for 5 min at 4°C. The supernatants were collected and washed 4 times with 4 volumes of water-saturated diethyl ether. The aqueous phase was dried up in a SpeedVac model Savant AS290 concentrator and resuspended in 180 µl water. cAMP concentrations were determined as described [29]. cAMP levels were also measured in the absence of phophodiesterase inhibitors. Finally, Δ[cAMP] (pmol/10^9^ platelets) represents increases of cAMP levels relative to the basal concentration measured on each experimental point.

### Measurement of the Levels of 12(S)-HETE by Immunoassay

Washed human platelets at 1×10^9^ cells/ml were aliquoted in 500 µl and placed before treatment into the aggregometer in a siliconized glass cuvettes warmed at 37°C and stirred. Cells were preincubated with 25 µM NDGA or vehicle for 5 min and subsequently were treated with 500 µM ATP or vehicle for 2 min at 37°C. Finally, cells were stimulated with 0.025 U/ml thrombin for 10 minutes at 37°C in the presence of 1 mM CaCl_2_. After the stimulation, 2 volumes of methanol were added and the samples were stored at −20°C. To measure 12(S)-HETE, 50 µl supernatants of stimulated platelets were evaporated under a stream of N_2_, and the residue was resuspended with 250 µl of ice-cold phosphate buffer saline. 12(S)-HETE levels were measured using an EIA kit according to the manufacturer’s instructions (Assay Designs, Ann Arbor, MI).

### Statistical Analysis

Results are expressed as means ± s.e.m. of the number of experiments indicated. Statistical significance between various conditions was assessed with Student’s *t* test.

## Results

### ATP Inhibits Platelet Aggregation Mediated by Thrombin

**Figure 1:**
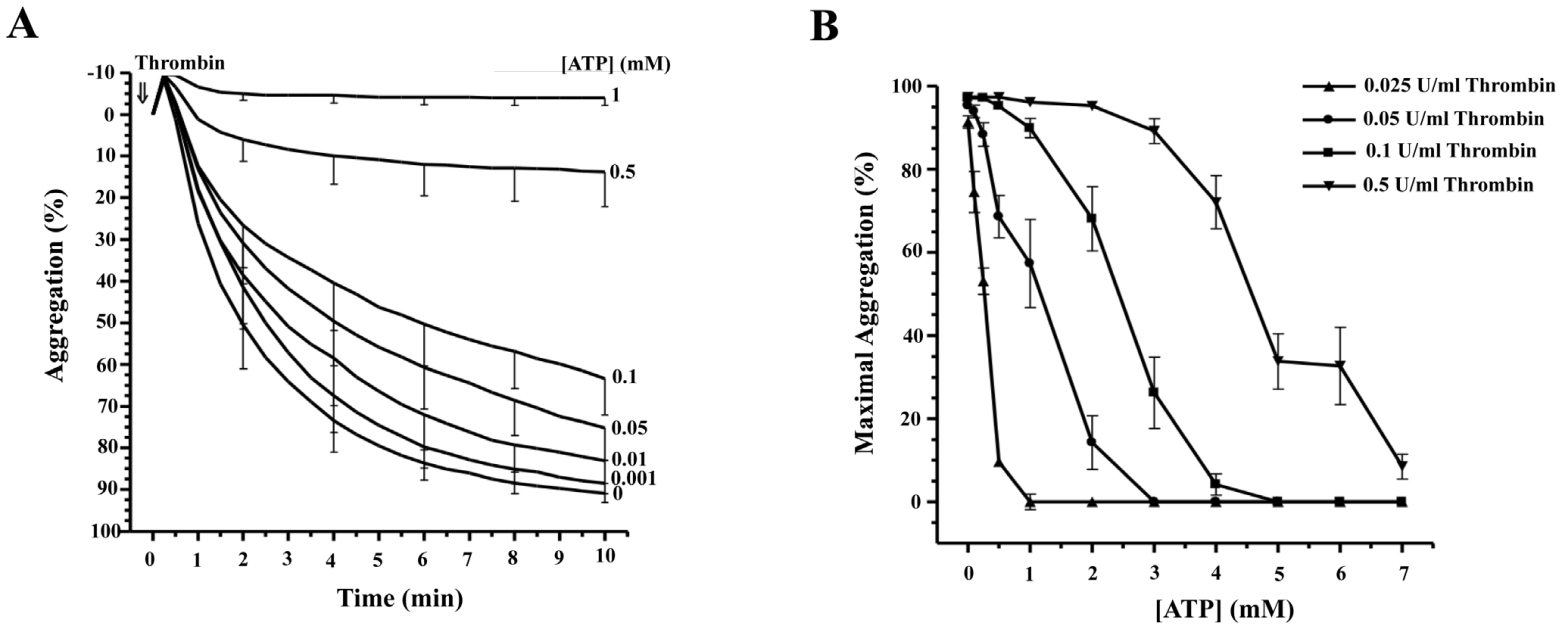
Inhibition of thrombin-induced platelet aggregation by ATP. Washed human platelets were preincubated with different concentrations of ATP or vehicle for 2 min. Then, cells were stimulated with (A) 0.025 U/ml thrombin (⇓, time = 0 and (B) with different thrombin concentrations: 0.025 U/ml (▴), 0.05 U/ml (♦), 0.1 U/ml (▪) or 0.5 U/ml (▾). The aggregation was measured for 10 min. (A) Values represent the average curves (%, means ± s.e.m) of 6 experiments with different platelet preparations. (B) Values represent the extent of aggregation at 10 minutes (%, means ± s.e.m) of 6 experiments with different platelet preparations. Error bars are omitted when smaller than symbols.

Washed human platelets were incubated with different concentrations of ATP (from 1 µM to 1 mM) for 2 min and were then stimulated with 0.025 U/ml thrombin for 2, 4, 6 8 and 10 min. As shown in Figure 1A, ATP concentrations, higher than 10 µM inhibited both phases of platelet aggregation in response to thrombin, the primary response-rate of aggregation, and the final response-maximal aggregation. The presence of 1 mM Mg^2+^ partially reversed the inhibition of TIPA mediated by ATP (Table 1). At higher concentrations Mg^2+^ significantly inhibited by itself the extent of TIPA (data not shown). 1 mM Mg^2+^ increased the IC_50_ for ATP from ∼250 µM up to ∼1 mM. These results suggested that ATP-mediated inhibition of platelet aggregation was ATP^4−^ dependent. Additionally increasing the concentration of thrombin also shifted to the right the IC_50_ for ATP (1.2 mM and 4.6 mM at 0.05 U/ml and 0.5 U/ml thrombin, respectively) (Figure 1B).

**Table 1:** Effect of Mg^2+^ on ATP-mediated inhibition of TIPA.

	Maximal Aggregation (%)	
[ATP] (mM)	− Mg^2+^	+ Mg^2+^
0	91±2	90±2
0.001	88±2	85±5
0.01	83±5	83±5
0.1	63±9	77±5
0.5	14±8	66±6
1	0±0	46±6
2	0±0	0±0

Washed human platelets were preincubated with different concentrations of ATP for 2 min in the absence (−Mg^2+^) or in the presence (+Mg^2+^) of 1 mM MgCl_2_. Then, cells were stimulated with 0.025 U/ml thrombin. The aggregation was measured for 10 min. Values represent the average of maximal aggregation (% means±s.e.m) of 7 experiments with different platelet preparations.

**Figure 2:**
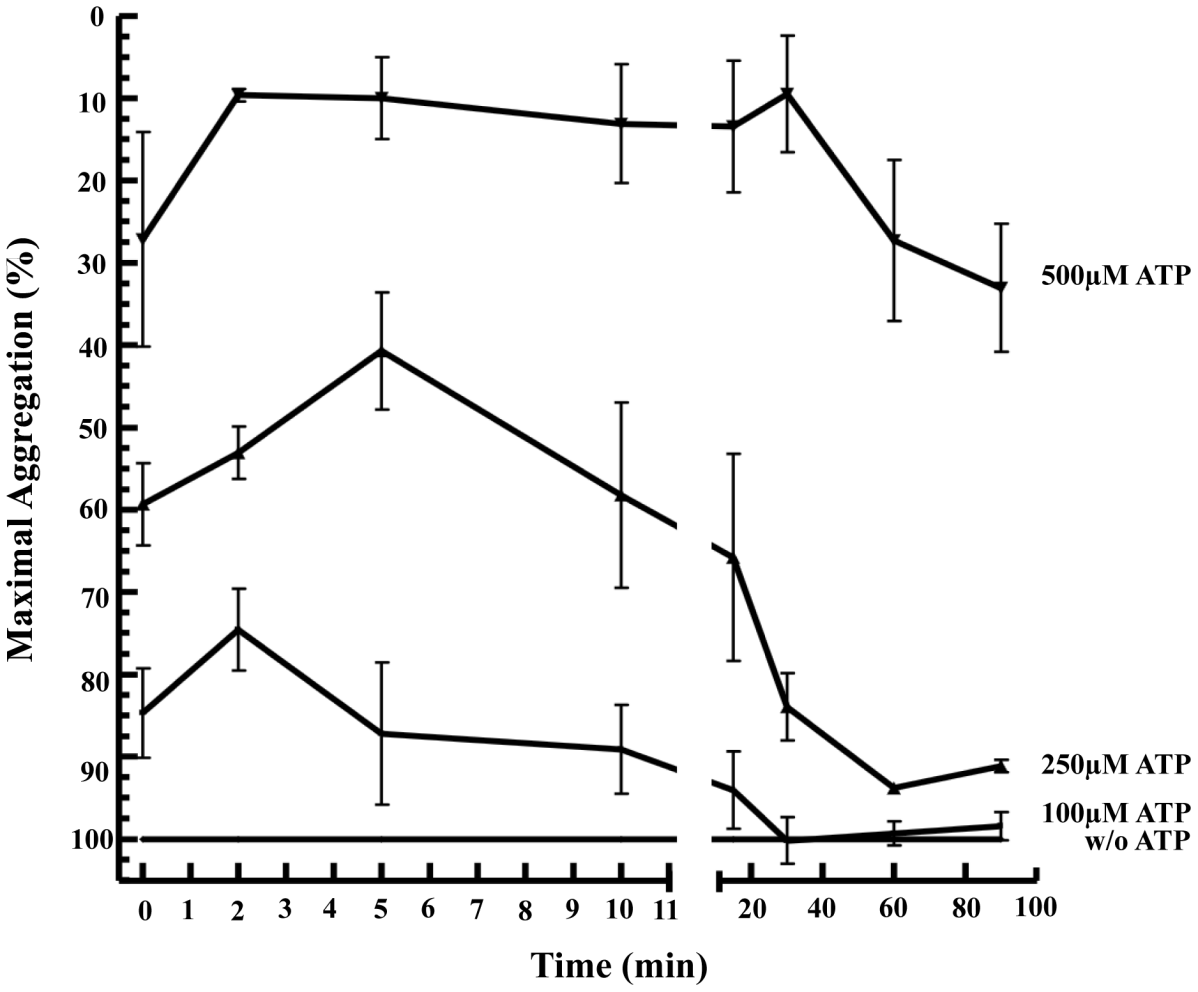
TIPA inhibition mediated by nucleotide is ATP time- and dose- dependent. Washed human platelets were incubated for various times (from 0 min to 90 min as indicated) with different ATP concentrations or vehicle. Then, cells were stimulated with 0.025 U/ml thrombin for 10 minutes. At 0 minutes ATP and thrombin were added simultaneously and maximal aggregation was measured after 10 min. Values represent the average of maximal aggregation (%, means ± s.e.m) of 3 experiments with different platelet preparations.

Next, we investigated whether ATP-mediated inhibition of TIPA was time-dependent. Washed human platelets were incubated with different concentrations of ATP (100, 250, 500 µM) for 0, 2, 5, 10, 20, 30, 60 and 90 minutes and were then stimulated with 0.025 U/ml thrombin. At time 0 platelets were exposed simultaneously to both ATP and thrombin. In all time points maximal aggregation was measured 10 minutes after addition of thrombin including time 0. As shown in Figure 2 the inhibition of TIPA mediated by nucleotide was very fast at all ATP concentrations (15±5%, 41±5%, 73±13% at 100, 250 and 500 µM ATP, respectively). This inhibitory effect slightly increased with incubation time (maximum at 2 min for 100 µM ATP and 5 min for 250 and 500 µM ATP) followed by a strong decrease in the inhibition carried out by ATP mainly at 100 and 250 µM, respectively (Figure 2).

### Effects of Selective Antagonists of ADP Receptors, P2Y_1_ and P2Y_12,_ on the TIPA Inhibition Induced by ATP

**Figure 3:**
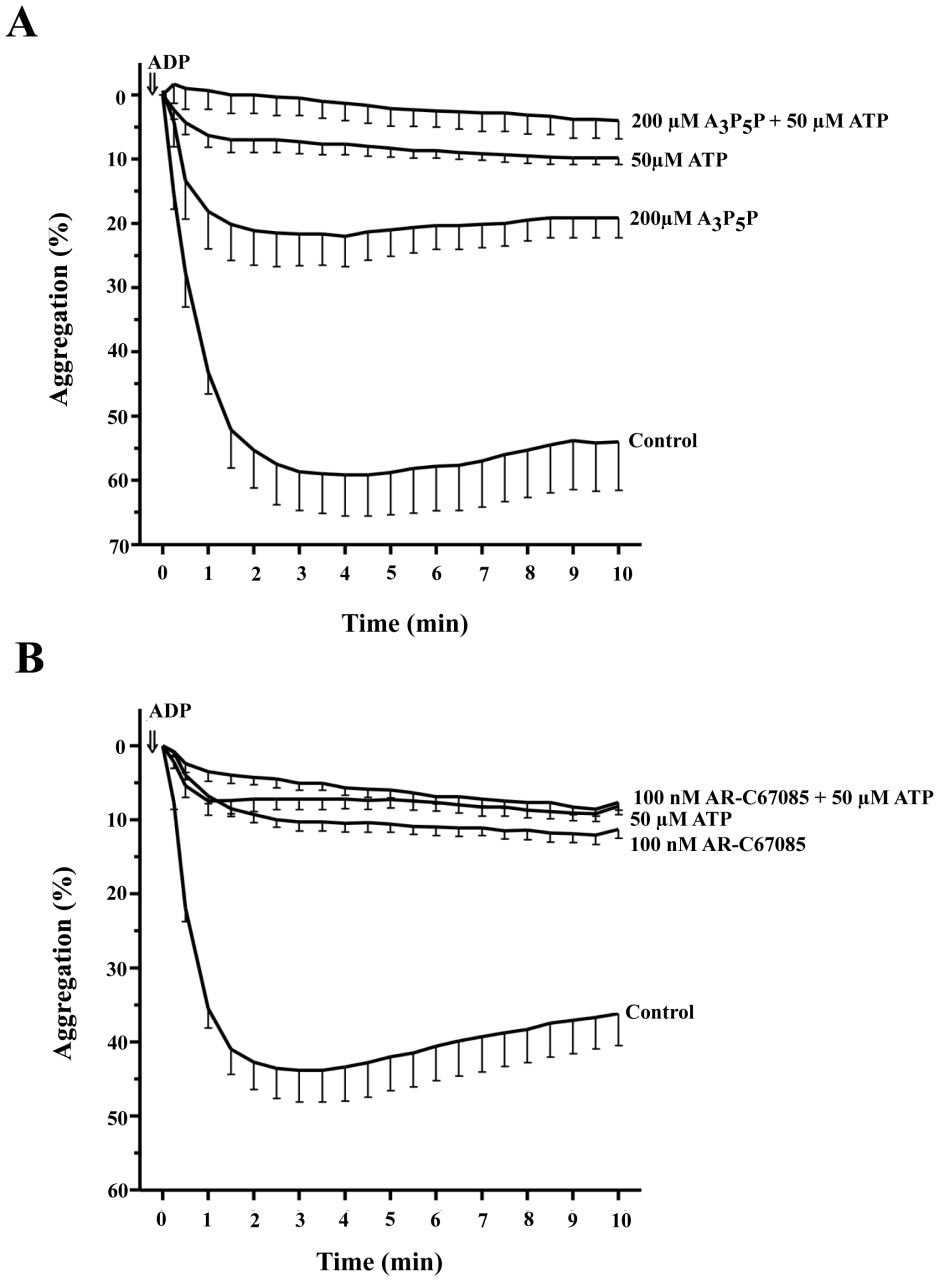
Effect of A_3_P_5_P and AR-C67085 on ADP-induced platelet aggregation. Washed human platelets were preincubated with (A) 200 µM A_3_P_5_P or vehicle for 10 min and (B) with 100 nM AR-C67085 or vehicle for 10 min. Then, cells were incubated with 50 µM ATP or vehicle for 2 min. Finally, cells were stimulated with 10 µM ADP (⇓, time = 0) in the presence of 0.3 mg/ml fibrinogen (added 1 minute before ADP). The assay was performed in the presence of 1 mM CaCl_2_. The aggregation was measured for 10 min and values represent the average curves of aggregation (%, means ± s.e.m) of 5 experiments with different platelet preparations.

**Figure 4:**
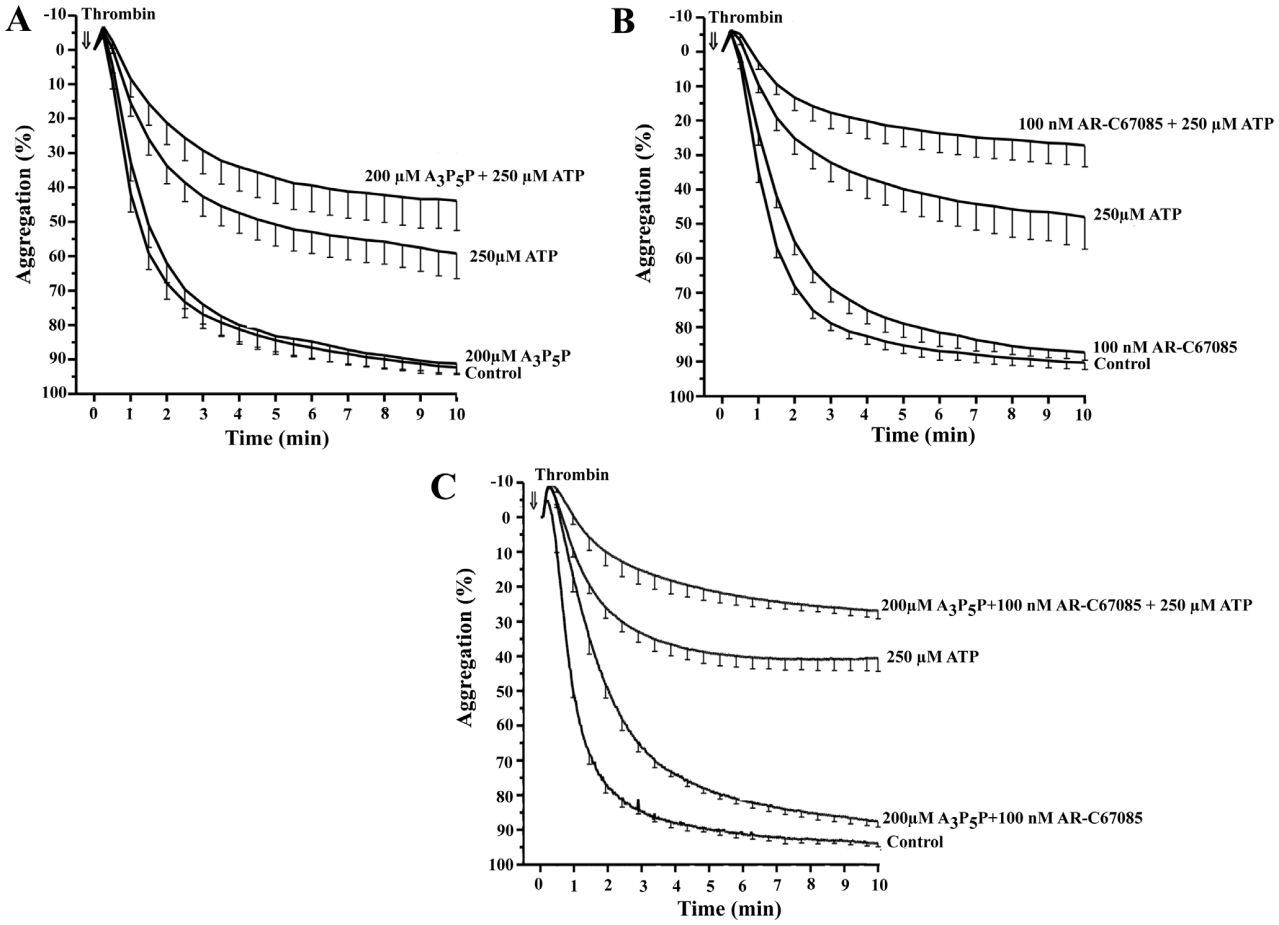
Effect of ADP receptor antagonists on the TIPA inhibition mediated by ATP. Washed human platelets were preincubated with (A) 200 µM A_3_P_5_P or vehicle for 10 min, (B) 100 nM AR-C67085 or vehicle for 10 min and (C) 200 µM A_3_P_5_P +100 nM AR-C67085 or vehicle for 10 min. Then, cells were incubated in the presence of ATP or vehicle for 2 min. Finally, cells were stimulated with 0.025 U/ml thrombin (⇓, time = 0 ). The aggregation was measured for 10 minutes and values represent the average curves of aggregation (%, means ± s.e.m) of 5 experiments with different platelet preparations.

It is widely accepted that extracellular ATP is a competitive antagonist of ADP-induced platelet activation [19]. To examine the potential implication of ADP receptors in the ATP-mediated inhibition of TIPA, we studied the effect of A_3_P_5_P, a selective antagonist for P2Y_1_ receptor and 2-propylthio-β,γ-difluoromethylene-D-ATP (AR-C67085), a structural analogue of ATP and a selective antagonist for P2Y_12_ receptor. Washed human platelets were preincubated or not with these compounds followed by incubation in the presence or absence of ATP and finally stimulated with 10 µM ADP. As shown in Figure 3 (A,B) ADP-induced platelet aggregation and this aggregation was strongly blocked by both P2Y selective antagonists (64% for 200 µM A_3_P_5_P; n = 3, P<0.01 and 68% for 100 nM AR-C67085, n = 5, P<0.01) and by 50 µM ATP (79%, n = 5, P<0.001). The effect of ATP on this response was marginally increased by co-incubation with either A_3_P_5_P or AR-C67085 (n = 5, P>0.05). Similar approach was developed to study the effects of these antagonists on the thrombin response. In this case, neither A_3_P_5_P nor AR-C67085 significantly modified the platelet aggregation induced by thrombin (Figure 4 A,B). However, AR-C67085 slightly increased (23±10%) the inhibitory effect produced by 250 µM ATP (Figure 4B). Next, the combination of both compounds had a small inhibitory effect on the rate and extent of aggregation (6±1%) on thrombin-stimulated platelets (Figure 4C). While the pretreatment of platelets with both compounds slightly increased (14±5%) the inhibitory effect induced by 250 µM ATP (Figure 4C).

### Effect of ADA and Apyrase on the TIPA Inhibition Induced by ATP

**Figure 5:**
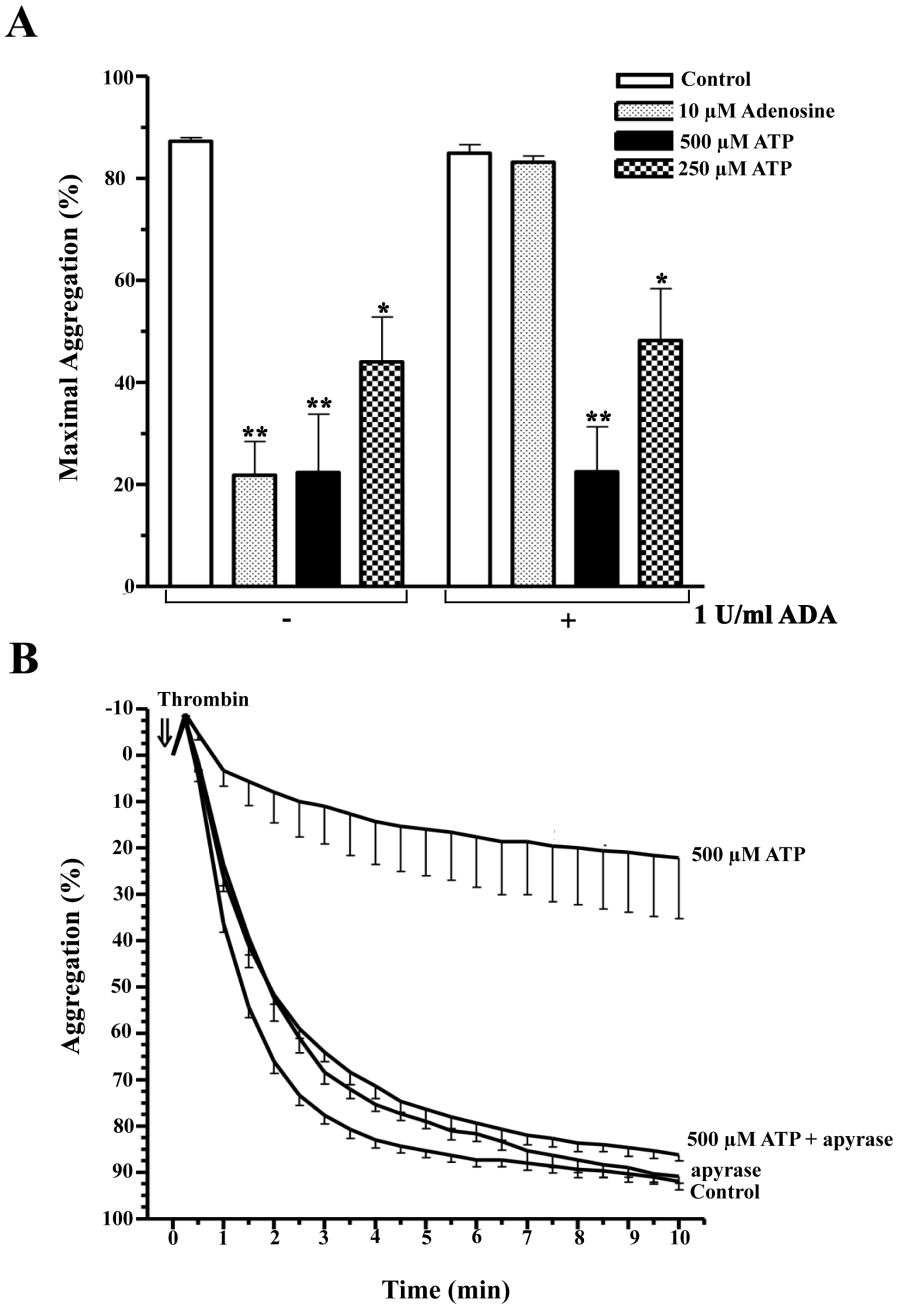
Effect of ADA and apyrase on the ATP-mediated inhibition of TIPA. (A) Washed human platelets were pretreated with 1 U/ml adenosine deaminase (+ ADA) or vehicle (- ADA) for 2 min. Cells were then incubated with 10 µM adenosine, 250 µM ATP, 500 µM ATP or vehicle (Control) for 2 min, and subsequently, stimulated with 0.025 U/ml thrombin (⇓, time = 0). Histogram represent the values of maximal aggregation (%, means ± s.e.m) of 3 experiments with different platelet preparations. *P<0.05, **P<0.01. (B) Washed human platelets were pretreated with 500 µM ATP or vehicle for 2 min, and subsequently with 0.5 U/ml apyrase or vehicle for 1 min. Finally, platelets were stimulated with 0.025 U/ml thrombin. The aggregation was measured for 10 min and values represent the average curves of aggregation (%, means ± s.e.m) of 3 experiments with different platelet preparations.

ATP effect on TIPA could be explained by the fast ATP hydrolysis to generate first AMP and subsequently adenosine, a well-known TIPA inhibitor. To test this possibility we examined the effect of ADA on our experimental system. As shown in Figure 5A, 1 U/ml ADA totally prevented TIPA inhibition induced by 10 µM adenosine (Fig. 5A, bars 2 and 6) in cells exposed to 0.025 U/ml thrombin. However, ADA was unable to modify the inhibitory effect of ATP on TIPA, both at 500 as 250 µM ATP (Fig. 5A, bars 3 and 7 and 4 and 8).

Further, to confirm that ATP itself was responsible for TIPA inhibition, we examined the effect of apyrase on platelet aggregation. Apyrase alone had no effect on TIPA (Figure 5B). Moreover, apyrase in the presence of ATP abolished the inhibitory effect induced by this nucleotide on platelet aggregation. The inhibition was reversed by 70% compared to platelets treated with thrombin and ATP in the absence of apyrase (Figure 5B). These results suggest that the presence of ATP in the incubation medium is required to observe the inhibitory effect on thrombin induced platelet aggregation.

### ATP Inhibits Second Messengers Generation Controlled by Thrombin

**Figure 6:**
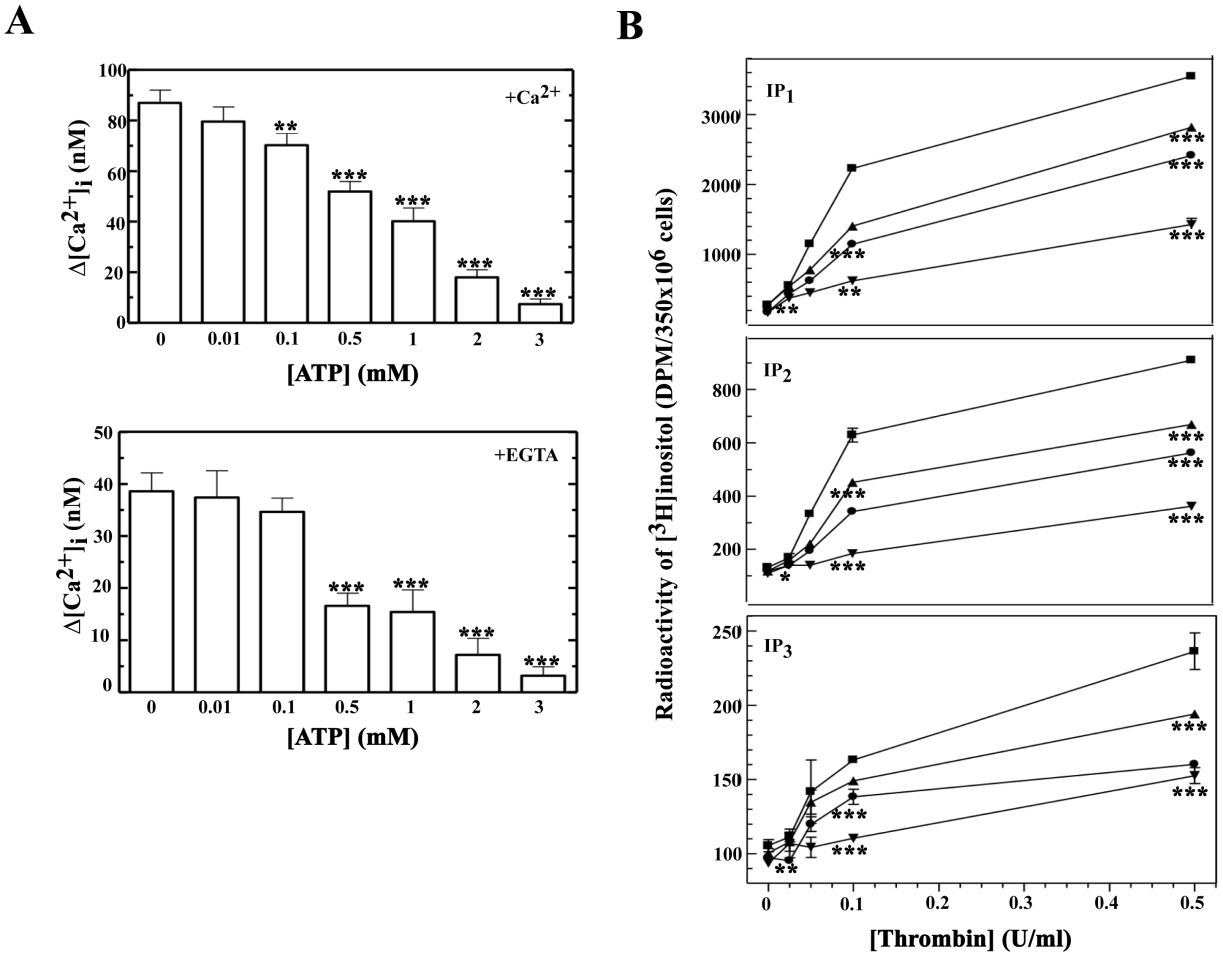
Effect of ATP on the thrombin-induced [Ca^2+^]_i_ mobilization and inositol phosphate generation. (A) Fura 2-loaded human platelets were incubated for 2 min with different concentrations of ATP (from 0 to 3 mM) in the presence (upper panel) or in the absence of 1 mM CaCl_2_ (lower panel). Then, cells were stimulated with 0.025 U/ml thrombin and the increase in [Ca^2+^]_i_ was measured for 90 s. The results are expressed as the variation of the [Ca^2+^]_i_ (Δ [Ca^2+^]_i_) measured before the addition of thrombin (basal level) and after the addition of thrombin (the maximum increase in [Ca^2+^]_i_). Histograms represent the means ± s.e.m of 5 experiments with different platelet preparations. In unstimulated cells the basal level in the presence of external calcium was 41±1 nM and in the presence of EGTA was 30±1 nM. In both cases, ATP did not affect the basal levels. **P<0.01, ***P<0.001. (B) [^3^H]Inositol-labeled platelets were incubated for 2 min with 0.5 (▴), 1 (•), 3 mM (▾) ATP or vehicle (▪). Then, cells were stimulated with different concentrations of thrombin (0, 0.025, 0.05, 0.1 and 0.5 U/ml) in the presence of 1 mM CaCl_2_. The reaction was stopped 10 min after the stimulation. Data are the means ± s.d. of one experiment performed in triplicate and representative of 2 other experiments. Inositol monophosphate (upper panel, IP_1_), inositol bisphosphate (middle panel, IP_2_), and inositol trisphosphate (lower panel, IP_3_) were separated by anion-exchange chromatography on a Dowex AG1-X8 column. Error bars are omitted when they are smaller than symbol. *P<0.05, **P<0.01, ***P<0.001.

Activation of platelets by thrombin involves phospholipase C (PLC) activation and is accompanied by an increase in [Ca^2+^]_i_ mobilization and cation entry from the extracellular environment [5]. In order to decipher the mechanism by which ATP inhibited platelet aggregation, we studied the effect of ATP on thrombin-induced [Ca^2+^]_i_ release in platelets. As shown in Figure 6A (upper panel), thrombin-induced robust intracellular calcium mobilization, from a baseline of 41 nM raise to 128 nM giving a maximum increase of 87±5 nM, however, this response was progressively and significantly inhibited by ATP in a dose dependent-manner. Maximal inhibition occurred in the presence of 3 mM ATP. Next, we studied the contribution of calcium from intracellular stores. To this end, the extracellular calcium was chelated with 2 mM EGTA. In these conditions thrombin induced a significant increase in intracellular calcium concentration (38±3 nM). This response was also blocked by ATP in a dose dependent-manner (Figure 6B, lower panel). Furthermore, ATP did not affect the basal [Ca^2+^]_i_ neither in the presence nor in the absence of extracellular calcium. These results suggested that ATP interfered negatively with early signals controlled by thrombin in the platelet aggregation process.

A key element in early signalling is the PLC, which mediates inositol phosphate break-down to generate IP3, DAG and [Ca^2+^]_i_. In order to investigate whether ATP blocked PLC activation mediated by thrombin, we examined inositol phosphate generation in platelets. As shown in Figure 6B, ATP inhibited significantly in a dose dependent-manner inositol monophosphate (IP_1_), bisphosphate (IP_2_) and trisphosphate (IP_3_) generation induced by all thrombin concentrations tested (from 0.025 U/ml to 0.5 U/ml). Moreover, maximal concentration of ATP (3 mM) inhibited also basal levels of IP_1,_ IP_2,_ IP_3_ by 39, 17 and 11%, respectively. Taken together these results indicate that ATP blocks platelet aggregation induced by thrombin and this process should require PLC activation to induce inositol phosphate generation and calcium mobilization.

### Effect of ATP on cAMP Generation in Human Platelets

**Figure 7:**
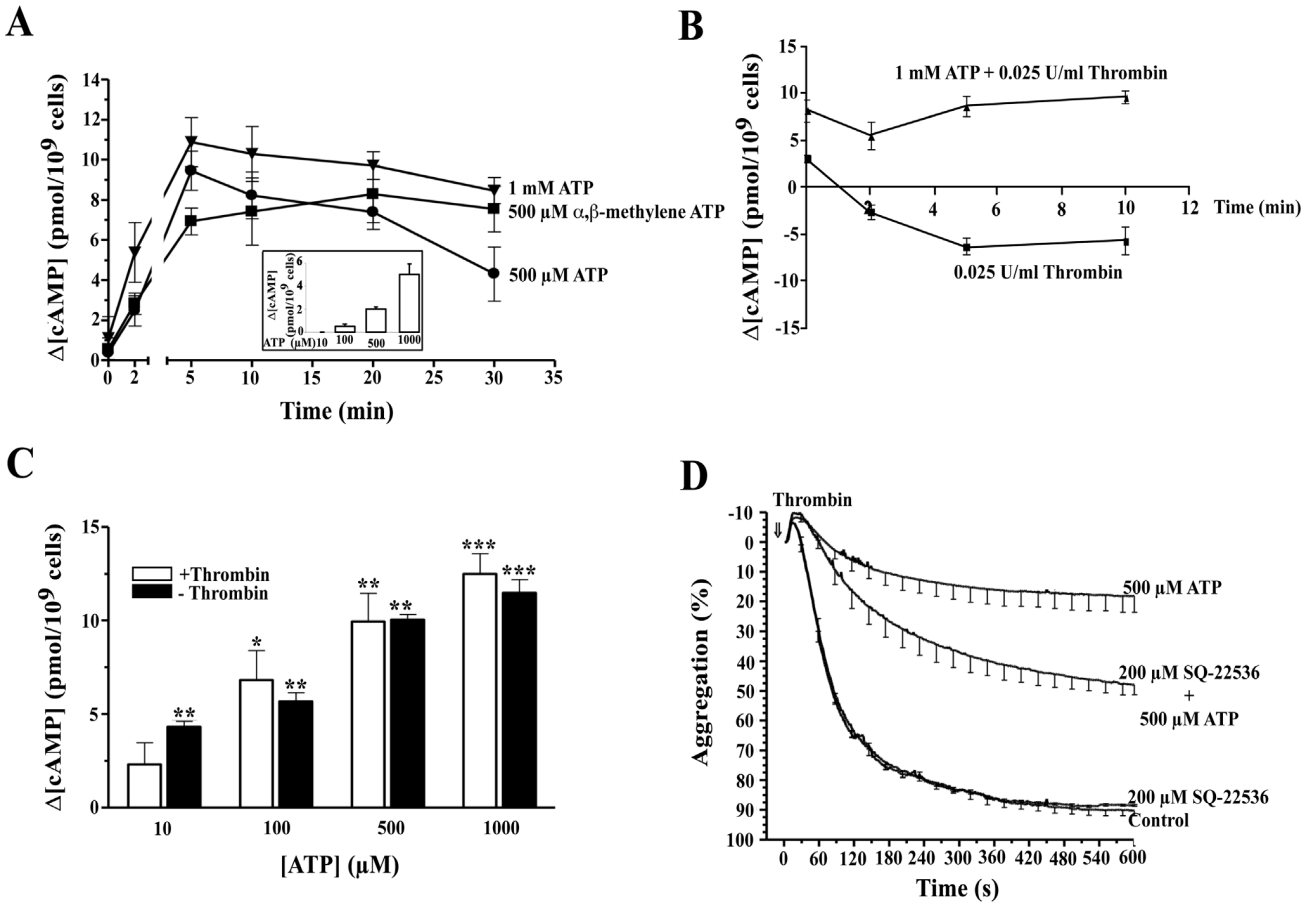
Effect of ATP on the cAMP levels in human platelets. (A) Washed human platelets were preincubated with a mixture of phosphodiesterase inhibitors for 5 min at 37°C, as indicated in Materials and Methods. Then, cells were incubated with 500 µM (•) or 1 mM (▾) ATP, (▪) 500 µM α,β-methylene ATP or vehicle for 2, 5, 10, 20 and 30 min. Inset, Washed human platelets were incubated with different concentrations of ATP for 5 min at 37°C in the absence of phosphodiesterase inhibitors. The reactions were stopped by addition of ice-cold TCA (10% final concentration). The cAMP levels were determined as described in Materials and Methods. Data represent the means ± s.e.m of 5 (A) and 3 (inset) experiments with different platelets preparations, performed in duplicate and assayed in triplicate. (B) Washed human platelets were preincubated with a mixture of phosphodiesterase inhibitors for 5 min at 37°C, as indicated in Materials and Methods. Then, cells were incubated with or without 1 mM ATP for 5 min at 37°C. Finally, cells were estimulated with 0.025 U/ml for 0, 2, 5 and 10 min in the presence of 1 mM CaCl_2_. The reaction was stopped by addition of ice-cold TCA (10% final concentration). The cAMP levels were determined as described in Materials and Methods. Data are the means ± s.e.m of 5 experiments with different platelets preparations, performed in duplicate and assayed in triplicate. (C) Washed human platelets were also preincubated with a mixture of phosphodiesterase inhibitors for 5 min followed by incubation in the presence of various concentrations of ATP or vehicle for 5 min and finally stimulated with 0.025 U/ml thrombin or vehicle for 5 min in the presence of 1 mM CaCl_2_. The reaction was stopped by addition of ice-cold TCA (10% final concentration). The cAMP levels were determined as described in Materials and Methods. Data are the means ± s.e.m of 5 experiments with different platelets preparations, performed in duplicate and assayed in triplicate. *P<0.05, **P<0.01, ***P<0.001. (D) Washed human platelets were preincubated with 200 µM SQ-22536 or vehicle for 2 min. Then, cells were incubated with 500 µM ATP or vehicle for additionally 2 min. Finally, cells were stimulated with 0.025 U/ml thrombin (⇓, time = 0). The aggregation was measured for 10 min and values represent the average curves of aggregation (%, mean±s.e.m) of 4 experiments with different platelet preparations.

In order to investigate the involvement of cyclic nucleotides and cyclic nucleotide-dependent protein kinases in the ATP-mediated inhibition of platelet responses, first we tested the guanylate cyclase inhibitor, ODQ which abolish the inhibitory effect of 10 µM SNP, (a nitric oxide donor that activates guanylate cyclase) on TIPA [26], [30]. This compound did not prevent the platelet aggregation inhibition induced by 500 µM ATP suggesting that guanylate cyclase/cGMP pathway was not involved in this process (data not shown). Moreover, we tested an adenylate cyclase (AC) inhibitor, SQ-22536 even when this AC inhibitor did not modify the basal TIPA, this AC inhibitor was able to partially block the inhibition induced by ATP over TIPA (Figure 7D). Next, we studied the effect of ATP on the cAMP accumulation in human platelets. First, to avoid cAMP degradation, we performed these experiments in the presence of different phosphodiesterase inhibitors, as described in Materials and Methods. As shown in Figure 7A, ATP and its non-metabolizable analog, α,β-methylene ATP, increased cAMP levels in a time-dependent manner reaching the maximun cAMP concentration after 5 min (9±1, 11±1 and 7±1 pmol/10^9^ cells for 500 µM ATP, 1 mM ATP and 500 µM α,β-methylene ATP respectively, n = 5, P<0.01). Longer incubations (up to 30 min) significantly reduced the increases in cAMP levels induced by 500 µM ATP (4±1 pmol/10^9^ cells, n = 5, P<0.05) but did not affect the response to 1 mM ATP or α,β-methylene ATP (n = 5, P>0.05). Then, we examined whether cAMP accumulation could be detected in the absence of phosphodiesterase inhibitors. To do this, washed human platelets were treated with different concentrations of ATP for 5 minutes and cAMP levels were measured as described in Materials and Methods. As shown in the inset of Figure 7 A, ATP induced an increase of intracellular cAMP levels in a dose-dependent manner. This increment were about 2 and 5 for 500 µM and 1 mM ATP respectively, whereas for the same concentrations of ATP in platelets which were pretreated with phosphodiesterase inhibitors the increments were about 9.5 and 11 (Figure 7A and inset). Nevertheless in the absence of inhibitors, the increment of cAMP was undetectable for 10 µM ATP and weakly detectable for 100 µM ATP, while at the same concentrations of ATP in the presence of phosphodiesterase inhibitors the increments were about 4.8 and 5.4 for 10 and 100 µM ATP respectively (Figure 7A inset compared to Figure 7C, first and second solid bars). Since, cAMP signal was amplified by using the cocktail of phophodiesterase inhibitors, we decided to perform the other experiments related with the intracellular cAMP detection in the presence of phosphodiesterase inhibitors.

Next, we studied the effect of ATP on cAMP levels in thrombin-stimulated human platelets. Consistent with the results obtained by Kim et al. [31], we also found that 0.025 U/mL thrombin reduced basal cAMP levels in a time-dependent manner (−6±1 pmol/10^9^ cells with respect to basal levels after 5 min, n = 5, P<0.01) (Figure 7B). However, when platelets were preincubated with 1 mM ATP for 2 minutes, followed by stimulation with thrombin (0, 2, 5 and 10 minutes), ATP not only blocked the effects of thrombin on the decreased of intracellular cAMP, but also ATP did the reverse effect, increasing cAMP to a value of 9 pmol/10^9^ cells (Figure 7B). Nevertheless, thrombin did not modify the cAMP accumulation in response to different concentrations of ATP (Figure 7C). These results suggested that ATP inhibition over agonist-induced platelet aggregation required AC/cAMP pathway.

**Figure 8:**
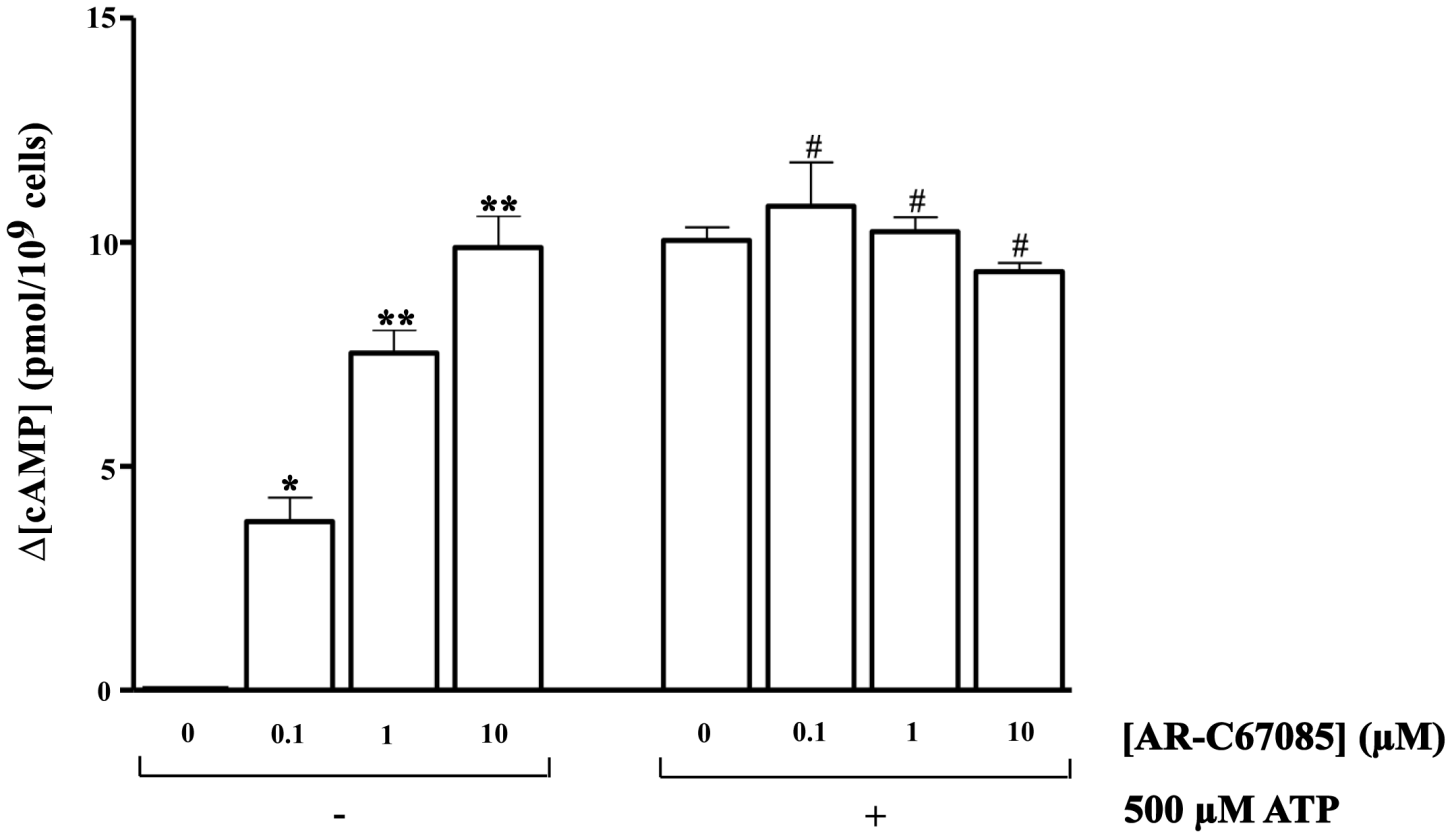
Effect of AR-C67085 concentrations on the cAMP levels in thrombin stimulated human platelets. Washed human platelets were preincubated with a mixture of phosphodiesterase inhibitors for 5 min. Then, cells were incubated in the presence of various concentrations of AR-C67085 (0.1, 1, 10 µM) or vehicle for 10 min and subsequently, were incubated in the presence of ATP (500 µM) or vehicle for 2 min. Finally, cells were stimulated with 0.025 U/ml thrombin for 5 min in the presence of 1 mM CaCl_2_. The reaction was stopped by the addition of ice-cold TCA (10% final concentration). The cAMP levels were determined as described in Experimental Procedures. Data are the means ± s.e.m of 4 experiments with different platelets preparations, performed in duplicate and assayed in triplicate. The increases in cAMP levels with respect to basal levels (Δ [cAMP], pmol/10^9^ platelets) are indicated. *P<0.5, **P<0.01 when compared to platelets not incubated with AR-C67085 in the absence of ATP and ^#^P>0.05 in the presence of ATP, respectively.

In a next set of experiments cAMP levels were measured in thrombin-stimulated platelets preincubated with AR-C67085 and treated or not with ATP. As shown in Figure 8, AR-C67085 increased cAMP levels in a dose-dependent manner, compared to control (platelet incubated with thrombin alone). Similar cAMP accumulation was observed in platelets incubated only in the presence of AR-C67085 (data not shown). When platelets were incubated first with different concentrations of AR-C67085 followed by 500 µM ATP, ATP also increased the cAMP intracellular levels in an AR-C67085 concentration independent manner. No potentiation or additive effect was observed by the combination of both P2Y_12_ receptor antagonists. Taken together these results suggested that ATP signalled through P2Y_12_ receptor in platelets to generate cAMP.

### NDGA Reverses the Platelet Aggregation Inhibition Mediated by ATP

**Figure 9:**
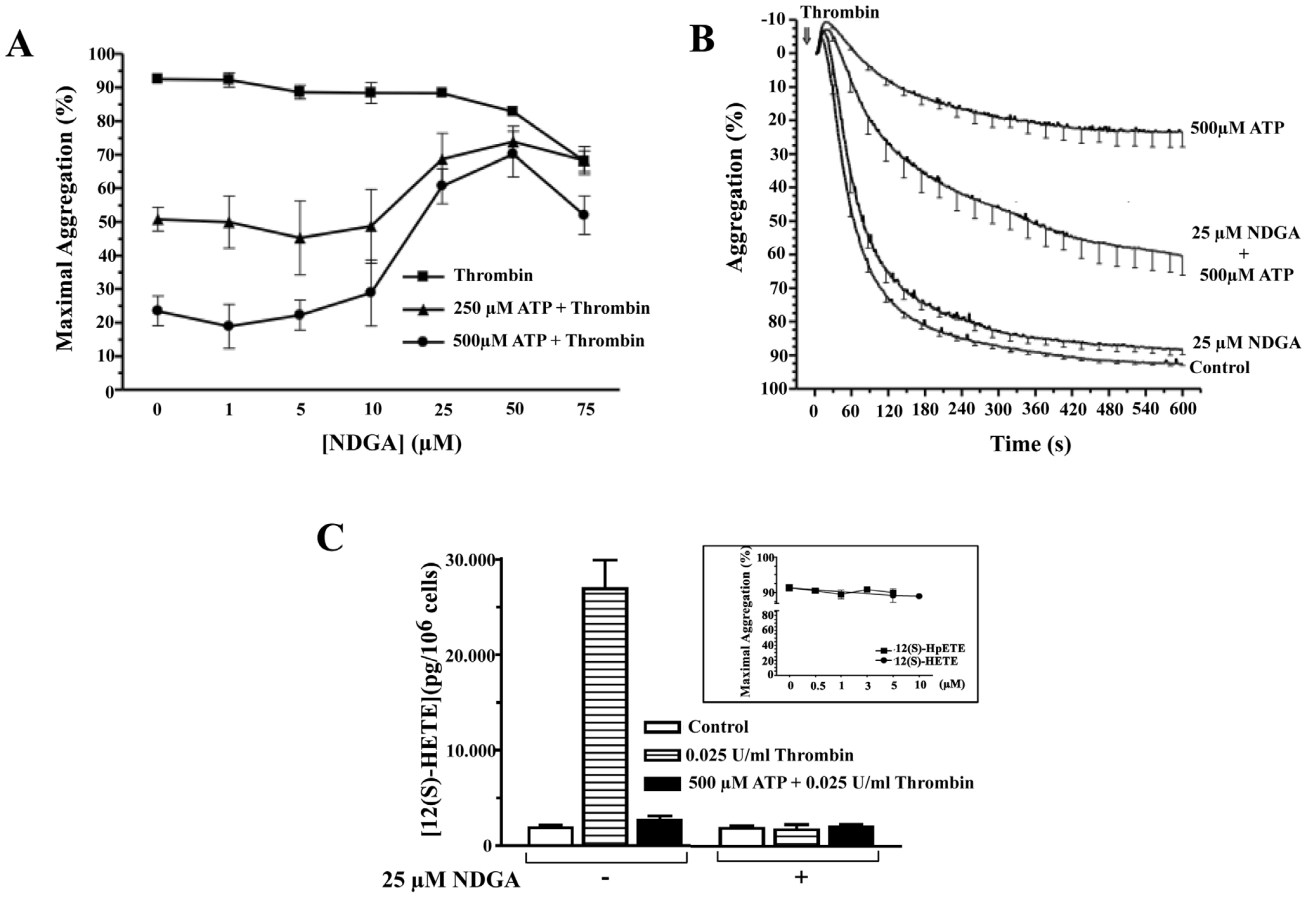
Effect of NDGA on the ATP-mediated inhibition of platelet aggregation and on 12(S) Lipoxigenase activity stimulated by thrombin.

12-LO and its metabolites are involved in the control of platelet response [32]. Based on this, we investigated the potential implication of this enzyme on the TIPA inhibition exerted by ATP. We preincubated platelets with different concentrations of NDGA (a potent lipoxygenases non-specific inhibitor) for 5 minutes, followed by 250 or 500 µM ATP and finally platelets were stimulated with thrombin. As shown in Figure 9A, the inhibition of platelet aggregation mediated by 250 and 500 µM ATP (45±3% and 74±5% respectively) was significantly reversed by NDGA at concentrations between 25 and 75 µM. Thereby in the presence of 25, 50 or 75 µM NDGA, the maximal inhibition was 23±7% (*P<0.05), 13±6% (**P<0.01) and 0±10% (**P<0.01), respectively. Resulting in a reversion of the inhibition induced by 250 µM ATP (50±10% (P<0.05), 73±13% (P<0.01) and 100±4% (P<0.01) at 25, 50 and 75 µM NDGA respectively, n = 4−9; Figure 9A). At 500 µM ATP and in the presence 25, 50 or 75 µM NDGA the reversion of inhibition was 60±12% (***P<0.001), 78±8% (**P<0.01) and 69±12% (*P<0.05) respectively (Figure 9A). We observed that 50 and 75 µM but not 25 µM NDGA inhibited the maximal platelet aggregation 10±1% and 26±3%, respectively (Figure 9A, solid squares), therefore we decided to use 25 µM NDGA in the next experiments.

To further confirm that concentrations of NDGA lower than 50 µM could significantly reverse the inhibition of TIPA mediated by ATP, we examined the effect of 25 µM NDGA on this process. As expected, 25 µM NDGA alone had no effect on TIPA (Figure 9B). Moreover, this concentration of inhibitor in the presence of ATP was able to reverse the aggregation blockage by 45% compared to platelets treated with thrombin and ATP in the absence of 25 µM NDGA (Figure 9B).

Next, we also examined the activity of 12-LO by measuring 12(S)-HETE generation. As shown in Figure 9C, thrombin stimulated 12(S)-HETE accumulation by more than 14-fold (second bar, hatched,) over control (first bar, empty bar). The generation of 12(S)-HETE due to LO activity was blocked when platelets were preincubated with 25 µM NDGA followed by thrombin stimulation (Figure 9C, fourth bar, hatched), however, in the same experimental conditions, NDGA did not inhibit platelet aggregation induced by thrombin (Figure 9B). Regarding the ATP, this nucleotide (500 µM) inhibited the thrombin-response over platelet aggregation (Figure 9B) and also reversed totally 12(S)-HETE concentration to basal level (Figure 9C, third and sixth bars, solids). Nevertheless, we found that in non-thrombin stimulated cells, extracellular ATP does not affect basal levels of 12 (S)-HETE (Figure 9C, first bar, empty). Taken together these results suggested that the signaling pathway related to LO and gluthatione peroxidase is crucial on the inhibition of the aggregation performed by ATP, since when it is blocked, the nucleotide effect is reversed around 60% (Figure 9B).

However, it was not clear the role of 12(S)-HETE in this process. To clarify this point, we examined directly the effects of two arachidonic acid metabolites, 12(S)-HETE and 12(S)-HpETE on aggregation induced by thrombin in our experimental conditions. Platelets were incubated with different concentrations of those metabolites for 2 min followed by 0.025 U/ml Thrombin in the presence of 1 mM CaCl_2_, maximal aggregation was determined at 10 minutes. As shown in the inset of the Figure 9C, results indicated that neither 12(S)HpTE nor 12(S)-HETE were able to modify the platelet aggregation led by thrombin.

**Figure 10:**
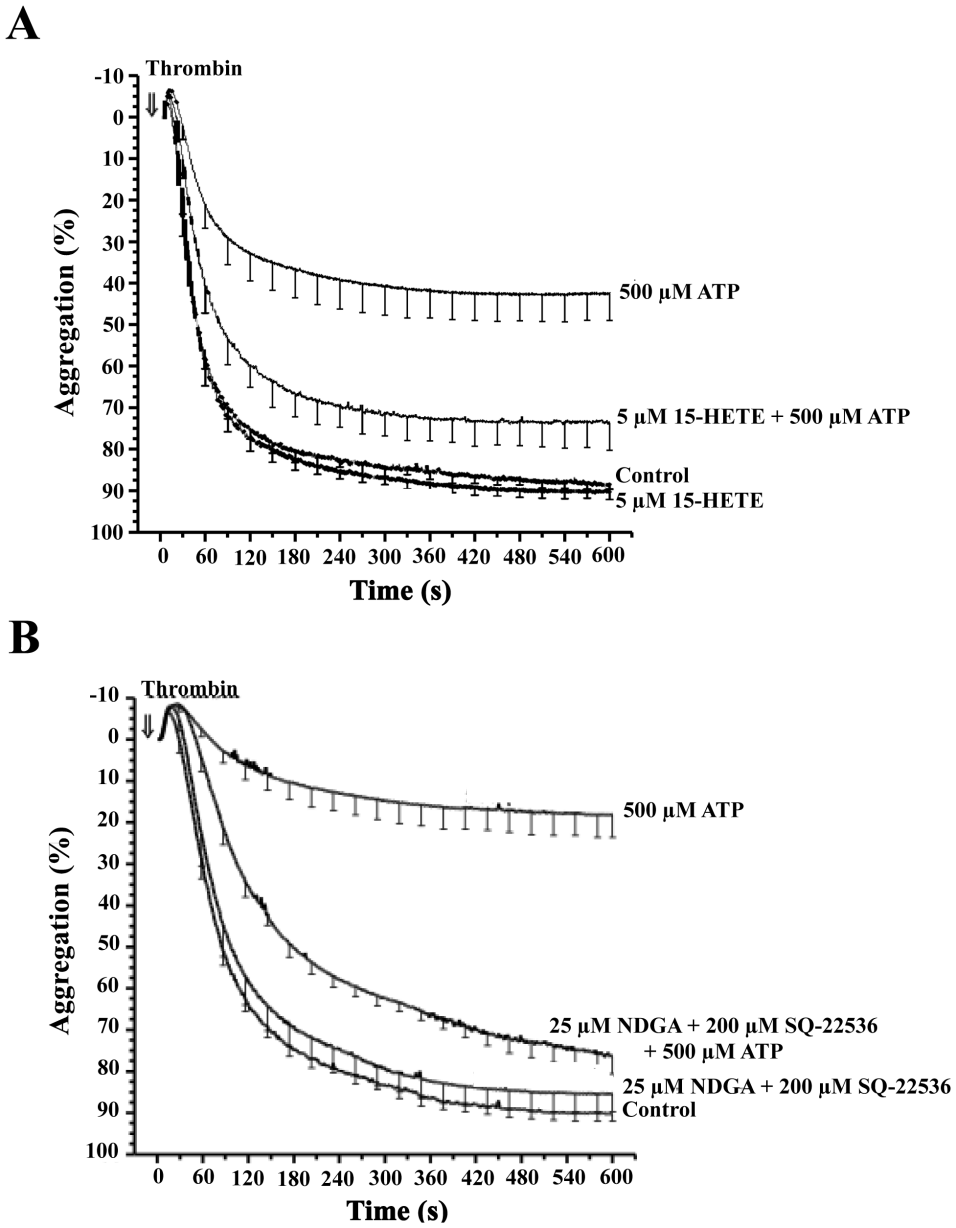
Effect of NDGA on the ATP-mediated inhibition of platelet aggregation. Washed human platelets were preincubated with (A) 5 µM 15(S)-HETE or vehicle for 2 min. Then cells were incubated in the presence of 500 µM ATP or vehicle for 2 min. Finally, cells were stimulated with 0.025 U/ml thrombin (⇓, time = 0). The aggregation was measured for 10 min. Values represent the extent of aggregation at 10 minutes (%, means±s.e.m) of 4 experiments with different platelet preparations. (B) cells were preincubated with 25 µM NDGA or vehicle for 5 min and with 200 µM SQ-22536 or vehicle for 2 min. Then, cells were incubated in the presence of 500 µM ATP or vehicle for 2 min. Finally, cells were stimulated with 0.025 U/ml thrombin (⇓, time = 0). The aggregation was measured for 10 min. Values represent the extent of aggregation at 10 minutes (%, means±s.e.m) of 6 experiments with different platelet preparations.

To investigate whether the effect of NDGA on ATP-mediated inhibition of TIPA was due to some interference in the cycloxygenase route, we tested the effect of 15(S)-HETE, a specific inhibitor of platelet 12-LO without any effect on cycloxygenase [33]. 15(S)-HETE did not significantly affect platelet aggregation in response to thrombin. Nevertheless, as well as NDGA, 5 µM 15(S)-HETE reversed the ATP-mediated inhibition of TIPA (Figure 10A, 67±10% (n = 4, P<0.01).

Finally, we studied the effect of both NDGA and SQ-22536 on ATP-mediated inhibition of platelet aggregation. Results show that combined incubation with NDGA and SQ-22536 did not affect TIPA significantly (n = 6, P>0.05; Figure 9D). However, ATP-mediated inhibition of TIPA was almost totally blocked. Inhibition of maximal aggregation by ATP alone was 76±5%, while it reached only 12±6% when both NDGA and SQ-22536 were present (Figure 10B).

Taken together these results suggested that ATP controlled intracellular antiaggregation signals through 12(S)-LO pathway.

## Discussion

In the present work we have shown that ATP were able to inhibit the aggregation of platelets in response to thrombin. The concentration of ATP in platelet granules is very high (between 100 and 400 mM) [34], [35] and the degranulation of platelets transiently increases the concentration of ATP in a range between 50 and 100 µM [36], [37]. Thus, the local concentration of ATP is probably much higher than the plasma concentration following platelet activation by thrombin [38]. Considering that the concentrations of ATP used in this work were very high, it could be argued that they would not correspond to physiological conditions, however, this assumption could be misleading, since it is recognized that the active form of ATP that regulates some P2X receptors activity is the free tetraionic form (ATP^4−^) [39], [40]. The complexation of ATP by divalent and monovalent cations reduces significantly the concentration of the active form of ATP. The major decrease of ATP^4−^ concentration occurs in the presence of Mg^2+^ [40]. In a Mg^2+^ free medium containing 1 mM ATP and in the presence of Ca^2+^, Na^+^ and K^+^, the concentrations of the active form of ATP are between 30 and 60 µM [40], [41]. These effective concentrations correlate within the range of concentrations of ATP found after platelet degranulations [36], [37].

Aggregation studies showed that the inhibition mediated by ATP was very rapid and dependent of this nucleotide, since pre-incubations with different concentrations of ADA (which completly block adenosine effects on platelet aggregation) had no effect on ATP-mediated inhibition of TIPA [42]. Platelets incubated with ATP beyond 15 minutes produced a decrease in the inhibition of the platelet aggregation. This reduction could be due either to a slow desensitization of the putative purinergic receptor involved in the response or to the action of ectonucleotidases or more likely of ectoapyrases bound to the platelet membrane in humans and rodents [42], [43]. In fact, the presence of apyrase in our experimental conditions corroborated that the observed inhibition on platelet aggregation was ATP dependent.

Besides aggregation, we found that ATP also inhibited the increase of the [Ca^2+^]_i_ induced by thrombin being this inhibition independent of the extracellular calcium, moreover, ATP also inhibited phosphatidyl inositol break-down at all concentrations of thrombin tested. These results suggest that PLC activity is affected by ATP. In agreement with that, Soslau et al. reported that extracellular ATP inhibited the calcium mobilization from intracellular pools by various agonists like collagen, thrombin or an analogue to thromboxane A_2_ [44], [45]. Similarly, we have described a mechanism independent of calcium influx, in which calcium increases induced by carbachol can be blunted by the activation of a P2X receptor with low affinity for ATP [46]. However, this inhibitory effect of ATP on intracellular calcium increases is controversial, it has also been reported that the ATP released from activated platelets induces intracellular calcium increases through P2X_1_ receptors [47]–[49]. These contrary results are probably due to differences in the experimental procedures. In order to prevent P2X_1_-receptor desensitization, platelet preparations were treated with apyrase [47]–[49]. While in our conditions apyrase was not used, this absence pointed out that P2X_1_ receptor would not be involved in this early signalling due to this receptor desensitizes in milliseconds without the presence of apyrase [20], [48], [50]–[52].

Many compounds exert their inhibition on TIPA through an increase in basal levels of cyclic nucleotides such as prostaglandins and NO donors [26], [53], [54]. ATP and α,β-methylene ATP produced a dose- and time-dependent increase in cAMP concentration. The increase in cAMP was rapid and transient, especially in the presence of ATP. This kinetic was probably due to the decrease of ATP concentration by ATP-specific ecto-apyrases. ODQ, a guanylate-cyclase inhibitor, did not alter the effect of ATP, suggesting that the increase in cAMP was not the result of the inhibition of the cyclic nucleotide phosphodiesterase type 3 by cGMP [53]. SQ-22536, an AC inhibitor, partially blocked the inhibition exerted by ATP on TIPA. According with that, Soslau et al. using 2′,5′-dideoxyadenosine as an AC inhibitor, reported that this compound partially reversed ATP-mediated inhibition of collagen-induced aggregation [55].

The levels of cAMP in platelets play a key role in the control of the activation/aggregation by thrombin. Eigenthaler et al. estimated that intracellular cAMP concentration in unstimulated washed platelets was 4.4 µM, similar to the concentration of cAMP binding sites of PKA [56]. Slight increases or decreases in these cAMP levels are sufficient to regulate in a positive or negative manner the activity of PKA and consequently the platelets are relaxed or activated [53], [57].

Thrombin reduces cAMP concentrations below the basal levels through the ADP secreted from dense granules in thrombin activated platelets [16], [31], [45]. ATP had the opposite effect, increasing cAMP levels independently of the presence of thrombin. When platelets were incubated at the same time with ATP and AR-C67085, a specific antagonist of P2Y_12_ receptors, AR-C67085 also increased in a dose-dependent manner the cAMP levels with a maximum increase at 10 µM. No potentiation or additive effect was observed when AR-C67085 was added to a maximal concentration of ATP, suggesting that ATP was acting on P2Y_12_ receptors with low affinity [58].

It has been reported that ADP contributes to platelet aggregation in response to low concentrations of thrombin [58]. However, we observed that apyrase did not significantly modify maximum aggregation induced by 0.025 U/ml thrombin. This is consistent with the results obtained by Ishii-Watabe et al. who reported that apyrase did not affect aggregation induced by 0.1 U/ml thrombin but fully inhibited the plasmin-induced aggregation [59]. The pharmacological inhibition of P2Y_1_ receptors with the antagonist A_3_P_5_P or N6-methyl-2′-deoxyadenosine-3′,5′-bisphosphate (MRS2179) had no effect on TIPA and it had no additional effects in combination with AR-C69931, an antagonist of P2Y_12_ [16]. These results confirmed those obtained from P2Y_1_ receptor-null mice, in which the absence of expression of these receptors did not affect TIPA [60]. The blockade of P2Y_12_ receptors with AR-C67085 did not inhibit thrombin-induced irreversible platelet aggregation; it slightly inhibited both phases of platelet aggregation, the primary response-rate of aggregation and the final response-maximal aggregation in response to 0.025 U/ml thrombin. Inhibition of maximal aggregation was reached at 1 µM, the contribution of this receptor to platelet aggregation was estimated about ∼12% in our conditions. Similar results were obtained in studies made on whole blood aggregation [16]. These results reveal that the inhibition of TIPA by ATP just cannot be explained by competitive inhibition at ADP receptor levels. In summary, we found that ATP increased cAMP levels by regulating negatively the P2Y_12_ receptor activation mediated by ADP and inhibiting platelet activation/aggregation induced by thrombin.

Extracellular nucleotides such as ATP, GTP and AMP are involved in the activation of cyclooxygenase and 12-LO in washed human platelets [24]. The arachidonic acid released by phospholipase A_2_ is a substrate of 12-LO for the production of 12(S)-HETE in platelets [61]. We have observed that ATP by itself did not affect phospholipase A_2_ activity (not shown), furthermore thrombin increases the 12(S)-HETE levels [62]. This eicosatetraeonic acid derivative modulates the platelet activity and pathological thrombus in acute coronary syndromes [63]. Regarding to platelet aggregation, it has been found that 12(S)-HETE enhances this process mediated by agonists such as thrombin by decreasing cAMP levels [63]–[65]. However, the inhibition of the 12-LO activity by either NDGA or 15(S)-HETE, a more specific 12-LO inhibitor, did not affect TIPA. Similar data have been obtained in platelets from 12-LO knock-out mice [66]. ATP decreased 12(S)-HETE levels in thrombin-stimulated human platelets, and its inhibition on TIPA was reversed by blocking 12-LO. These results suggest that a metabolite of the AA/12-LO pathway mediated the inhibition of TIPA by ATP. Based on preliminary results showing that ATP does not modify thrombin stimulated 12(S)-HpETE formation (not shown), we hypothesize that a derivative of 12(S)-HpETE different to 12(S)-HETE could be responsible for the antiaggregant effect produced by ATP. In fact, there is evidence supporting the role of these metabolites as inhibitors of platelet aggregation induced by different agonists [67].

When both AC and 12-LO were simultaneously inactivated by SQ-22536 and NDGA respectively, ATP-mediated TIPA inhibition was almost completely abolished. This effect was additive not synergistic, suggesting the presence of two different and independent mechanisms whereby ATP exerted its inhibition. According to with this, platelet incubated either with NDGA or 15(S)-HETE did not lead to any significant change in cAMP levels (data not shown), indicating that 12-LO inhibition did not interfere with the effect of ATP on AC. Conversely the inhibition of AC with SQ-22536 did not affect thrombin stimulated 12(S)-HETE formation or ATP-mediated inhibition of this hydroxylipid (data not shown).

In conclusion, we have shown that the inhibition of either AC or 12-LO pathway reverses ATP-mediated inhibition of TIPA. Furthermore, ATP exerts its inhibitory effect over TIPA at least through two different and independent signalling routes: 1) by regulating AC activity; the intracellular cAMP levels negatively affects the intracellular signalling controlled by thrombin, and also interferes with the ADP/P2Y_12_-receptor pathways led by thrombin. In addition, we postulate that the rapid effect of ATP on TIPA inhibition could also be originated by a direct allosteric interaction between the ATP and a P2 receptor different to P2Y_12._ Although the molecular mechanism remains to be elucidated; 2) through another unknown molecular mechanism that involves to the 12-LO pathway. Future studies will focus on the P2 receptor identification as well as the characterization of the molecular events leading to the cooperation between cAMP and 12-LO pathways to inhibit the platelet aggregation induced by thrombin.
